# Swiss Practice Recommendations for Chronic Prurigo Including Prurigo Nodularis

**DOI:** 10.1111/ijd.70533

**Published:** 2026-06-18

**Authors:** Simon M. Mueller, Helmut Beltraminelli, Wolf‐Henning Boehncke, Maximilian Böhm, Antonio Cozzio, Peter Häusermann, Ahmad Jalili, Antonios G. A. Kolios, Claudia Lang, Eva Loos, Christoph Schlapbach, Curdin Conrad

**Affiliations:** ^1^ Department of Dermatology University Hospital Basel Basel Switzerland; ^2^ Department of Dermatology Ente Ospedaliero Cantonale (EOC) Bellinzona Switzerland; ^3^ Division of Dermatology and Venereology Geneva University Hospitals Geneva Switzerland; ^4^ Division of Dermatology and Allergology HOCH Health Ostschweiz St Gallen Switzerland; ^5^ Private Practice Basel Basel Switzerland; ^6^ Dermatology & Skin Care Clinic Buochs Switzerland; ^7^ Department of Medicine Hospital Uster Uster Switzerland; ^8^ Department of Dermatology University of Zürich Zurich Switzerland; ^9^ Immunologie‐Zentrum Zurich Switzerland; ^10^ Department of Psychosomatic Medicine University Hospital Basel Basel Switzerland; ^11^ Department of Dermatology Inselspital, Bern University Hospital, University of Bern Bern Switzerland; ^12^ Department of Dermatology Lausanne University Hospital CHUV and University of Lausanne Lausanne Switzerland

**Keywords:** chronic prurigo, chronic pruritus, practice recommendations, prurigo nodularis, Swiss experts

## Abstract

Management of chronic prurigo (CPG), including prurigo nodularis (PN), is challenging due to its chronic, often treatment‐refractory course, high disease burden, and increasing complexity of therapies. International guidelines are partly outdated and may not fully reflect country‐specific considerations. Our aim was to develop practice recommendations for the diagnosis and treatment of CPG. Twelve hospital‐ and practice‐based experts formulated recommendations following a standardized literature review and structured expert consensus process, with recommendations voted on until ≥ 75% agreement was reached. Recommendation levels were guided by the GRADE methodology but adapted to allow practical, differentiated guidance, including the additional “Can be considered” category. Recommendations address history taking, laboratory testing, biopsy, questionnaires, general measures, pharmacological and non‐pharmacological therapies. “We recommend” applies to topical corticosteroids, narrow‐band ultraviolet B (NB‐UVB), dupilumab, and nemolizumab; “We suggest” to topical calcineurin inhibitors, zinc oxide dressings, cryotherapy, intralesional corticosteroids, gabapentinoids, antidepressants, methotrexate, ciclosporin, μ‐opioid receptor antagonists, Janus kinase inhibitors, and cognitive behavioral techniques; and “Can be considered” to capsaicin, ultraviolet A1 (UVA1), psoralen plus ultraviolet A (PUVA), and sedating antihistamines for sleep. No recommendation could be made for azathioprine, non‐sedating antihistamines, κ‐opioid receptor agonists, retinoids, neurokinin‐1 receptor antagonists, thalidomide, or lenalidomide. These recommendations provide comprehensive, up‐to‐date guidance for CPG, supporting clinicians in Switzerland and internationally.

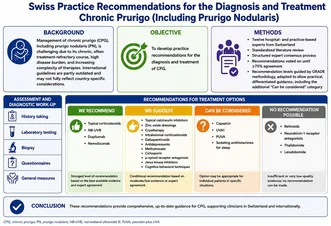

## Graphical abstract

Chronic prurigo, including prurigo nodularis, is a chronic, often treatment‐refractory condition characterized by persistent pruritus and chronic scratch lesions. Advances in the understanding of its underlying pathophysiology have enabled the development of novel targeted therapies; however, management remains challenging due to the expanding range of therapeutic options. Swiss experts have developed practical recommendations for diagnostic work‐up and a personalized treatment approach, including vulnerable patient populations, that are not only applicable in Switzerland but also generalisable to most other Western countries.

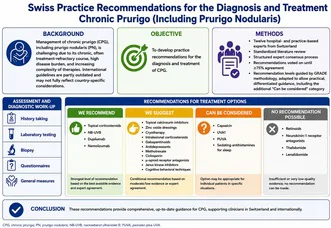

## 1 Introduction

Chronic prurigo (CPG) is a neuroimmune skin condition defined by (1) The presence of chronic pruritus (itch lasting ≥ 6 weeks), (2) A history and/or signs of repeated scratching, and (3) Multiple chronic scratch lesions that are either localized or generalized [1, 2]. The prevalence of CPG in Europe was reported to be approximately 30/100,000 population [3, 4, 5, 6], the most common causes are chronic dermatological conditions (e.g., atopic dermatitis), systemic diseases (including renal and hepatic dysfunction, neuropathy), and psychiatric comorbidities. However, in many cases no identifiable underlying disease can be found [1, 3, 4, 7, 8].

CPG is often associated with stress, sleep disturbance, anxiety, and depression, resulting in a significant reduction in quality of life [9, 10, 11]. According to an expert consensus paper, CPG is an umbrella term that encompasses several clinical subtypes, which are classified based on the predominant morphology of the lesions [1]. These subtypes include nodular (also referred to as “prurigo nodularis” [PN]), papular, plaque, umbilicated, and linear forms [1]. The authors of this recommendation are aware of the ongoing controversy surrounding the terminology of CPG versus PN, as no international consensus has yet been achieved. Given that most clinical studies have been published using the term PN, we have chosen to adopt the compromise title “chronic prurigo, including prurigo nodularis” for this recommendation, in alignment with the terminology used in the IFSI (International Forum for the Study of Itch) guideline [12].

PN is the most common and best‐studied subtype, driven by complex interactions among keratinocytes, resident and circulating immune cells, fibroblasts, skin vessels, and the peripheral and central nervous systems, leading to a self‐perpetuating itch–scratch cycle, fibrosis, and increased vascularization [13, 14, 15, 16]. PN lesions show infiltration of Th2, Th17, and Th22 cells, eosinophils, macrophages, and mast cells, with increased expression of interleukin (IL)‐4, IL‐13, IL‐17, IL‐22, and particularly IL‐31, which correlates directly with itch intensity [14, 17, 18].

In recent years, advances in the pathophysiological understanding of chronic prurigo have enabled the development of novel targeted therapies, yet the management remains challenging due to its chronic, often treatment‐refractory course, and the expanding spectrum of available treatment options. As the currently available international guideline on CPG has not been updated since 2020 [12] and does not specifically address important aspects such as personalized management and treatment in vulnerable populations, we have compiled the condensed practice recommendations presented here, which may be useful not only in Switzerland but also beyond.

## 2 Methods

On January 16 and April 3, 2025, 12 hospital‐ or practice‐based experts (11 senior dermatologists including leading clinicians of the five university hospitals in Switzerland and one psychologist) met to develop recommendations both for the diagnostic work‐up and treatment of CPG. All experts contributing to these recommendations practice in Switzerland. Nevertheless, this work aimed to develop recommendations that are not only applicable in Switzerland but also generalisable to most other Western countries. Switzerland‐specific reimbursement limitations for dupilumab and nemolizumab are outlined in the respective “Biologics” subchapter.

Based on a standardized non‐systematic literature search (see Appendix A) and expert opinion, recommendations were formulated and voted on. At least 75% of the votes had to be in favor to approve a recommendation. Recommendation levels (“*We recommend*”, “*We suggest*”, “*We cannot make a recommendation*”, “*We suggest against*”, “*We recommend against”*) defined by the GRADE (Grading of Recommendations, Assessment, Development, and Evaluation) working group [19] were applied. We additionally included the option “Can be considered” to allow for more differentiated recommendations. Final adjustments were made in October 2025 following nemolizumab's approval for PN in Switzerland.

## 3 Recommendations

### 3.1 History Taking

As summarized in Table 1, the following assessments are key when taking the medical history of patients with CPG:

1. **Pruritus:** Evaluate duration, distribution, intensity (0–10 mean Numeric Rating Scale [NRS] and/or Worst Itch NRS (WI‐NRS) during past 24 h and 7 days), and sensory quality (e.g., itching, burning, stinging, prickling). Identify potential triggers (e.g., stress, heat), alleviating factors (e.g., cooling, distraction), and B‐symptoms. This helps differentiate dermatological, systemic, neuropathic, and psychogenic causes. Itch‐related quality of life can be assessed with the ItchyQoL [20, 21, 22, 23] or, less suitable, with the Dermatology Life Quality Index (DLQI).
2. **Skin lesions and scratching behavior:** Determine whether primary skin lesions are present, their type, distribution, onset, and evolution. Evaluate scratching patterns to clarify whether pruritus arises on diseased or healthy skin and to guide habit‐reversal training.
3. **Medical history:** Review systemic diseases (e.g., renal, hepatic, endocrine, malignancies), current and past medications (including pruritogenic or antipruritic agents), psychiatric comorbidities, and family history of skin disorders to identify underlying or contributing factors.
4. **Sleep and psychiatric comorbidities:** Assess itch‐related sleep disturbance, depression, and anxiety, given their high prevalence in CPG patients and impact on quality of life. The Regensburg Insomnia Scale (RIS) has proven a suitable screening tool in patients with chronic pruritus [24], two short standardized questions may be used to screen for depression and anxiety [25]: For this purpose, we recommend using the Generalized Anxiety Disorder 2‐item (GAD‐2) [26, 27, 28], and Patient Health Questionnaire 2‐item (PHQ‐2) [29, 30, 31]. Be aware that delusional infestation may mimic CPG [32]; refer to psychiatrists' referral in case of suspected psychiatric comorbidity.

**TABLE 1:** Recommendations for history taking, diagnostic approach and assessment of the severity and activity of CPG. (* Modified according to Zeidler et al. Acta Derm Venereol. 2021 February 17;101(2)).

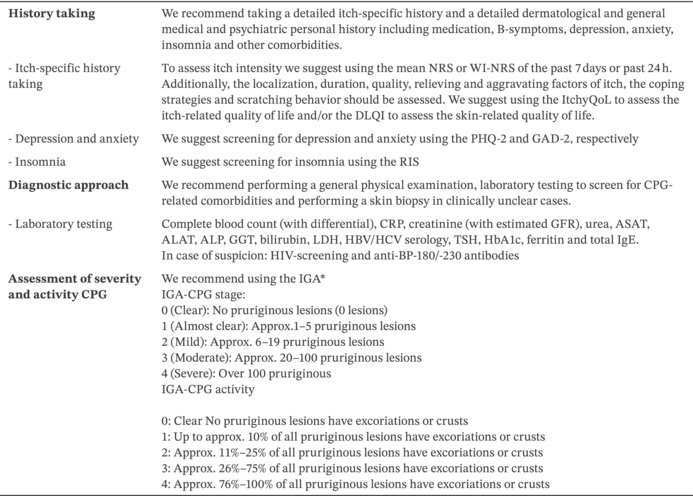

Abbreviations: ALAT, alanine aminotransferase; ALP, alkaline phosphatase; ASAT, aspartate aminotransferase; BP, blood pressure; CRP, C‐reactive protein; DLQI, Dermatology Life Quality Index; GAD‐2, generalized anxiety disorder 2‐item questionnaire; GFR, glomerular filtration rate; GGT, gamma‐glutamyl transferase; HbA1c, glycated hemoglobin; HBV, hepatitis B virus; HCV, hepatitis C virus; HIV, human immunodeficiency virus; IGA, investigator's global assessment; ItchQoL, itch quality of life; LDH, lactate dehydrogenase; PHQ‐2, Patient Health Questionnaire‐2; RIS, Regensburg Insomnia Scale; TSH, thyroid‐stimulating hormone; WI‐NRS, Worst Itch Numerical Rating Scale.

### 3.2 Diagnostic Work‐Up

Clinical assessment of the type and number of scratch lesions (e.g., nodules, papules, plaques, umbilicated, or linear) according to [33], as well as the presence of potential primary skin lesions that may indicate underlying or associated dermatological conditions, is essential for tailoring topical and systemic treatment.

For the assessment of disease severity and activity, the Investigator's Global Assessment (IGA) is the most used and recommended instrument (Table 1) [34]. In patients with underlying or concomitant active atopic dermatitis, severity assessment using the Eczema Area and Severity Index (EASI) is recommended, as it may support in‐label use of ciclosporin, dupilumab, nemolizumab, or Janus kinase inhibitors.

In addition, a general physical examination should be performed to identify lymphadenopathy and/or other organ pathology that may be related to CPG.

Basic laboratory testing is primarily recommended for patients with CPG of unclear origin as summarized in Table 1 (adapted with minor modifications from the IFSI guideline [12]).

A skin biopsy is indicated only in unclear cases and/or when specific diagnostic questions arise, such as prebullous bullous pemphigoid or small fiber neuropathy‐ the latter can be confirmed by reduced intraepidermal nerve fiber density using PGP9.5 immunostaining [35].

### 3.3 Treatment

As opposed to the IFSI guideline, this recommendation does not follow a 4‐step treatment ladder but a different approach as depicted in Figure 1, consisting of general measures that may apply to all CPG patients followed by a more personalized additional treatment based on three criteria:

1. **Disease severity** according to the IGA. Patients with moderate‐to‐severe CPG, defined by 20 lesions or more and a mean daily WI‐NRS ≥ 7/10 over the past 7 days, qualify for a systemic antiinflammatory treatment [36, 37].
2. **Level of recommendation** of the pharmacological and non‐pharmacological treatment options based on the IFSI guideline, our updated literature search, availability, and expert opinion.
3. **Individual suitability** for these treatment options. This is of particular importance in vulnerable patient groups, including the elderly, pregnant or breastfeeding women, cancer patients, and children. Our treatment recommendations for these patient groups are outlined in Figure 2.

**FIGURE 1:**
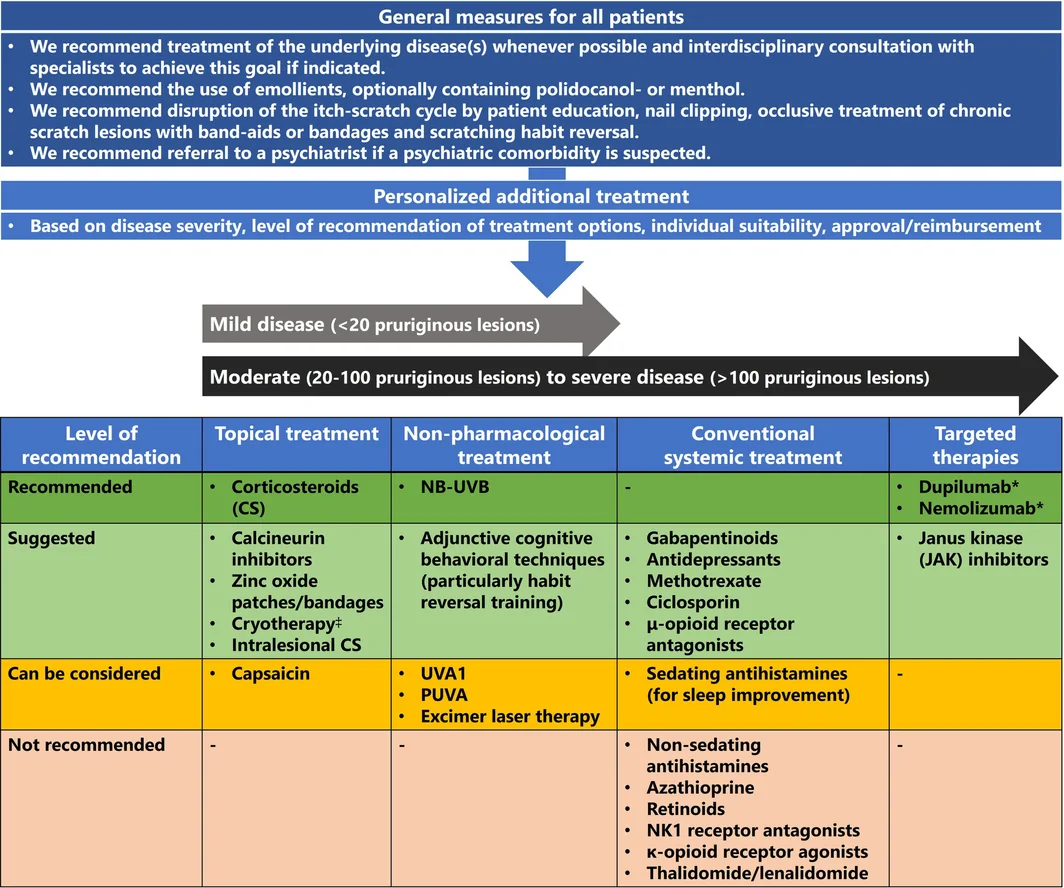
Treatment recommendations including general measures and additional personalized treatment according to disease severity and level of recommendation for the listed pharmacological and non‐pharmacological (NB‐UVB, UVA1, PUVA, excimer laser, cryotherapy) treatment options.* Dupilumab and nemolizumab are the only treatments approved for “PN.” ‡ Cryotherapy should be limited to few lesions and used cautiously in skin of color due to risk of dyspigmentation. NB‐UVB, narrowband ultraviolet B phototherapy; NK1, neurokinin‐1 receptor; PUVA, psoralen plus ultraviolet A phototherapy; UVA1, ultraviolet A1 phototherapy.

**FIGURE 2:**
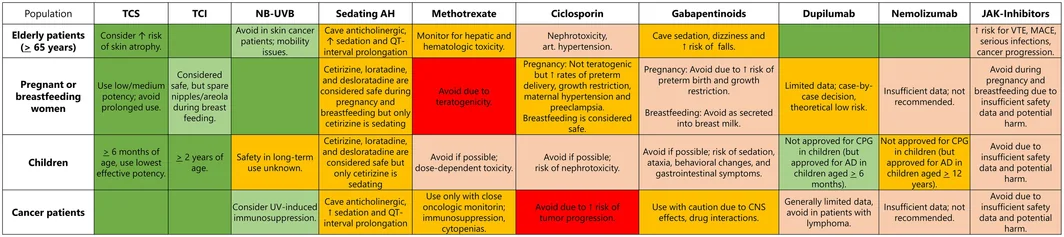
Treatment recommendations for the vulnerable populations elderly patients, pregnant or breastfeeding women, children and cancer patients. Color coding: Dark green: recommended; light green: suggested; orange: can be considered; light red: not recommended; dark red: contraindicated. AH, antihistamines; CNS, central nervous system; MACE, major cardiovascular events; NB‐UVB, narrow band UVB; TCI, topical calcineurin inhibitors; TCS, topical corticosteroids; VTE, venous thromboembolism.

#### 3.3.1 General Measures

All patients with CPG may benefit from general measures, including:

1. **Treatment of underlying conditions** whenever possible, with referral to other specialties if needed to achieve this goal.
2. **Daily use of emollients**, optionally containing antipruritic agents such as polidocanol (2%–5%) or menthol (1%–2%, cooling relief via TRPM8 activation) [38, 39].
3. **Disruption of the itch‐scratch cycle**.

For the latter, patient education should emphasize the proinflammatory effects of scratching, nails should be kept short, and practical strategies to cope with the urge to scratch should be provided, such as making fists, counting to 30, or scratching with the dorsal side of the hand or wearing cotton gloves to avoid skin damage from fingernails. Occlusive measures using band‐aids or bandages may also help to interrupt the itch‐scratch cycle. Particularly, zinc oxide patches/bandages have been shown to be highly effective in treating CPG in a Swiss randomized split‐body study [40], with similar results reported by a Swedish group [41].

#### 3.3.2 Topical Treatments

##### 3.3.2.1 Topical Corticosteroids (TCS) and Topical Calcineurin Inhibitors (TCI)

TCS are considered first‐line therapy for CPG due to their antiinflammatory and thereby indirect antipruritic effects [42, 43, 44]. Survey data indicate modest efficacy, which can be enhanced when used under occlusion [41]. Thick, treatment‐resistant lesions might benefit from intralesional TCS [45]. Limitations of TCS include the risk of skin atrophy with prolonged use and the lack of robust randomized controlled trial data specifically in CPG.

TCI (tacrolimus and pimecrolimus) may be less effective than TCS but can serve as steroid‐sparing agents, particularly in steroid‐sensitive areas or for long‐term management [42, 43, 46, 47]. Transient burning at the application site is mediated by activation of TRPV1 channels, which are also activated by capsaicin [46]. The level of evidence for TCI is limited to small studies and case series with variable outcomes [43, 47].

**We recommend TCS and suggest TCI on lesional skin in CPG for 7–14 days 1/day, thereafter 1–2/week as maintenance treatment if necessary. We suggest intralesional steroids (e.g., 3 infiltrations 3 weeks apart) in thick, treatment‐refractory lesions and zinc oxide patches/bandages in combination with or without TCS for 7–14 days**.

##### 3.3.2.2 Capsaicin

The results of clinical studies and systematic reviews support the use of capsaicin in concentrations between 0.025% and 0.3% in CPG, which may provide itch relief within days to weeks [43, 48]. Initial burning due to excitation of TRPV1‐expressing C‐fibers is common but not a prerequisite for subsequent desensitization and clinical benefit [43, 49]. Its use is generally reserved for localized, refractory lesions due to the need for frequent application (4–6 times daily) to achieve sustained benefit [43, 50]. Recurrence of itch is common after discontinuation, and comparative efficacy versus other topicals remains unknown [50].

**The use of topical capsaicin can be considered in localized forms of CPG.**

##### 3.3.2.3 Cryotherapy

Cryotherapy has occasionally been used as a destructive treatment option for CPG lesions; however, its role remains limited, with evidence restricted to small comparative studies and a few case reports [42, 51, 52, 53, 54]. Treatment protocols vary considerably with respect to the number of freeze–thaw cycles (typically 2–4), freezing duration (approximately 5–30 s), treatment frequency (single or repeated sessions), and the use of concomitant topical corticosteroids, precluding precise and consistent recommendations precise regarding the optimal application of this modality. Cryotherapy may be considered for isolated, treatment‐refractory nodules, but it is not suitable for widespread disease and lacks robust data supporting long‐term efficacy or safety. Potential adverse effects include pain, blistering, scarring, and dyspigmentation.

**We suggest cryotherapy (maximum of two freeze–thaw cycles with a freezing time of 10–15 s) for highly treatment‐refractory, localized lesions. In patients with skin of color, the risk of dyspigmentation should be carefully considered.**

#### 3.3.3 Systemic Treatments

##### 3.3.3.1 Antihistamines

Although systemic antihistamines are the most commonly used systemic treatment for CPG in Europe [55, 56, 57], evidence for their efficacy is very limited, as histamine is not a key itch mediator in CPG and controlled clinical trials are lacking. However, there is evidence that mast cells play a role in the pathophysiology of CPG [58, 59, 60], as they are increased in lesional skin and may contribute to neurogenic inflammation and pruritus. Consistent with this concept, treatment with the mast cell–depleting anti‐KIT antibody barzolvolimab showed clinically meaningful reductions in itch and improvements in skin lesions in a phase 1b study [61]. At present, only sedating H1 antihistamines should be used in selected patients mainly for their sleep‐promoting effects and not for a direct antipruritic benefit.

**We cannot make a recommendation for antihistamines in CPG. If a sedating effect is desired, first‐generation antihistamines can be considered.**

##### 3.3.3.2 Antidepressants

Antidepressants, including selective serotonin reuptake inhibitors (SSRIs; e.g., paroxetine, sertraline), tricyclics (e.g., amitriptyline) and tetracyclic mirtazapine, are used in CPG, particularly in patients with comorbid depression, anxiety, or sleep disturbance [43, 62, 63, 64]. Apart from their antidepressant, anxiolytic, and affect‐modulating effects, antidepressants may also reduce pruritus by modulating central and peripheral neurotransmitter pathways, amitriptyline and mirtazapine additionally have sleep‐promoting properties. However, evidence for antidepressants in CPG is limited, mainly from case series and small trials [43, 65]. If a sleep‐promoting rather than an antidepressant effect is desired, mirtazapine 7.5–15 mg/day may be considered. Adverse effects should be closely monitored, and patients with suspected psychiatric comorbidity should be referred to a psychiatrist [43, 65].

**We suggest the use of antidepressants (e.g., paroxetine, mirtazapine) in CPG. For sleep promotion without an antidepressant indication, mirtazapine 7.5–15 mg/day is suggested.**

##### 3.3.3.3 Gabapentinoids

Gabapentinoids (gabapentin, pregabalin) are mainly effective for neuropathic itch, a component that can also occur in CPG patients. Although systematic reviews support their use, the evidence remains low, as it is largely limited to small open‐label studies and case series [44, 66, 67, 68, 69]. Gabapentin is typically administered at gradually uptitrated doses ranging from 100 to 2400 mg/day, while pregabalin is used at doses of 75–300 mg/day. Dose adjustment in patients with renal impairment is mandatory, and the increased risk of falls, fractures, and altered mental status must be carefully considered, particularly in elderly patients and in those receiving concomitant opioid therapy.

**We suggest the use of gabapentinoids in CPG, especially when itch is burning, prickling, stinging, or painful, indicating a neuropathic component.**

##### 3.3.3.4 Opioids (μ‐Opioid Receptor Antagonists and κ‐Opioid Receptor Agonists)

μ‐Opioid receptor antagonists (e.g., naltrexone) and κ‐opioid receptor agonists (e.g., difelikefalin) have demonstrated antipruritic effects in uremic, cholestatic and neuropathic chronic pruritus by modulating central and peripheral itch pathways [65, 70]. However, the evidence specifically for CPG remains low: Oral naltrexone (50–150 mg/day) improved both itch and skin lesions in an uncontrolled open‐label study [71] and in case series [47, 72]. Oral nalbuphine, a μ‐opioid antagonist and κ‐opioid agonist, showed promising efficacy and safety in CPG, but in Switzerland it is only available as an injectable. The κ‐opioid receptor agonist difelikefalin is approved globally only for patients undergoing hemodialysis.

Opioid use is limited by adverse effects (e.g., nausea, insomnia, and gastrointestinal symptoms) and is suitable only in selected cases, particularly given the availability of more effective approved systemic therapies for CPG.

**We suggest the use of oral μ‐opioid receptor antagonists naltrexone in selected patients but cannot make a recommendation for κ‐opioid receptor agonists.**

##### 3.3.3.5 Conventional Systemic Antiinflammatory Therapies

Systemic anti‐inflammatory treatment of CPG is indicated in moderate‐to‐severe CPG. Vaccination status should be reviewed and updated before starting such therapy, with live vaccines given ≥ 4 weeks prior and inactivated vaccines ideally before therapy or with temporary drug holds to optimize immunogenicity.

Unless contraindicated, prior treatment with a conventional systemic therapy is a prerequisite for reimbursement of modern targeted therapies in most countries, including Switzerland.

The most up‐to‐date comparative evidence for ciclosporin, methotrexate, and azathioprine in CPG is limited to systematic reviews, small retrospective studies, and case series [43, 44, 69]. Ciclosporin (3–5 mg/kg/day) has demonstrated significant antipruritic effects and lesion improvement in case series; however, adverse effects such as hypertension and renal impairment are common and limit long‐term use [43, 69, 73, 74]. Notably, ciclosporin is approved for atopic dermatitis, which may overlap with CPG in some patients, thereby allowing for in‐label prescription.

Methotrexate also showed high overall response rates (mean dose 15 mg/week) after 3–12 months in a multicenter retrospective study with sustained clinical benefit but increased risks of hepatotoxicity and infection [43, 69, 74, 75, 76].

Evidence for azathioprine in CPG is anecdotal [69, 77], its tolerability may be limited by myelosuppression and gastrointestinal symptoms [78].

**We suggest using ciclosporin (for a maximum of 6 months) or methotrexate in CPG but cannot make a recommendation for the use of azathioprine.**

##### 3.3.3.6 Biologics

Dupilumab and nemolizumab are the most extensively studied therapies in CPG and, in many countries including Switzerland, currently the only agents approved for this indication.

In Switzerland, the limitation for reimbursement of these drugs is [37]:

Moderate‐to‐severe prurigo nodularis in patients aged ≥ 18 years, WI‐NRS ≥ 7 and ≥ 20 lesions, provided that topical corticosteroids, phototherapy (if available/indicated), and a systemic conventional immunosuppressant (excluding systemic steroids) used for at least 1 month were insufficient, contraindicated or not tolerated.

Additionally, increased itch intensity and/or pain, new papules, nodules, more erosions and crusts can indicate disease worsening on a conventional systemic drug that may prompt evaluation of a switch to a biologic. We suggest a treatment algorithm guided by the clinical response to dupilumab or nemolizumab, as shown in Figure 3.

**FIGURE 3:**
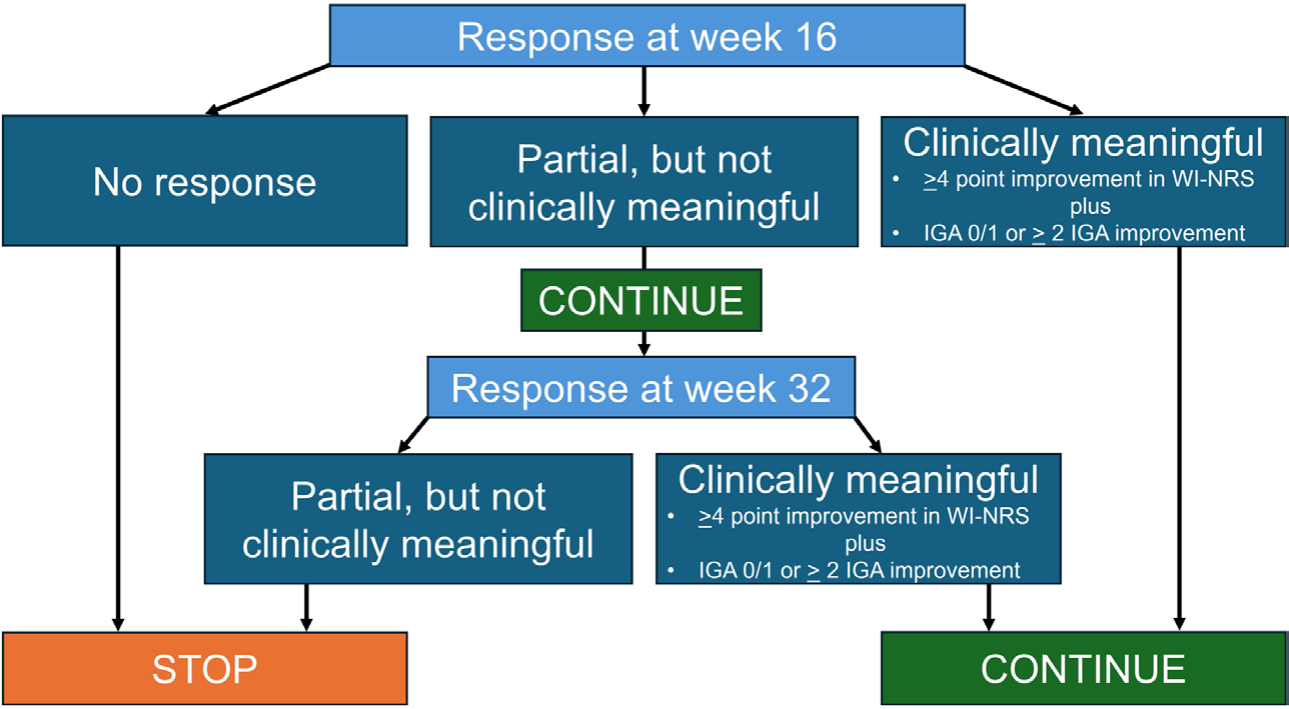
Recommended treatment strategy depending on the response to dupilumab or nemolizumab. WI‐NRS, Worst Itch Numerical Rating Scale; IGA, Investigator's Global Assessment.

##### 3.3.3.7 Dupilumab

Dupilumab targets interleukin‐4 receptor alpha subunit (IL‐4Rα), which is shared by both the IL‐4 and IL‐13 receptor complexes, thereby inhibiting signaling of the type 2 cytokines IL‐4 and IL‐13. It is administered subcutaneously 300 mg every 2 weeks. In the pivotal phase‐3 trials LIBERTY‐PN PRIME and PRIME 2 (concomitant use of TCS or TCI), the ≥ 4‐point improvement of the WI‐NRS was achieved by 59% (averaged from the two studies) of patients and clear or almost clear skin (IGA 0/1) by 46% at Week 24 [79, 80]. In a systematic review and meta‐analysis, dupilumab treatment was associated with a mean reduction in itch score of 5.76 points, along with a significant improvement in quality of life [81]. Long‐term data from pooled analyses and real‐world studies indicate that the majority of patients maintain clinically meaningful improvements in pruritus and skin lesions beyond 24 weeks, with sustained reductions in WI‐NRS and improvements in quality of life [82, 83, 84]. The safety profile of dupilumab in CPG is favorable with the most common adverse events being mild conjunctivitis and injection‐site reactions [79, 81, 83, 84, 85].

**We recommend the use of dupilumab in patients with moderate or severe CPG.**

##### 3.3.3.8 Nemolizumab

Nemolizumab blocks the IL‐31 receptor, which is expressed on various immune cells (e.g., Th2 lymphocytes, dendritic cells, eosinophils, and mast cells), pruriceptive nerve fibers, keratinocytes, and fibroblasts, resulting in beneficial effects on inflammation, pruritus, epidermal dysregulation, and dermal fibrosis [14, 86, 87]. In the pivotal OLYMPIA 1 [88] and 2 [89] trials, nemolizumab monotherapy achieved a ≥ 4‐point itch reduction in 57% of patients and an IGA 0/1 response in 32% at Week 16, with significant itch relief observed as early as day 2. These data and our clinical experience suggest faster itch reduction, but slower skin improvement compared with dupilumab, though no head‐to‐head data exist. This assumption is further supported by an indirect clinical trial comparison of dupilumab and nemolizumab, which suggested that dupilumab may provide greater lesional clearance at later time points, whereas nemolizumab may offer more rapid itch relief and reduced sleep disturbance [90]. In the OLYMPIA open‐label extension, the ≥ 4‐point itch reduction was reached by 92% and IGA 0/1 response by 74%, indicating full response may take longer than 16 weeks [91]. Nemolizumab was well tolerated, with mild‐to‐moderate headache, eczematous reactions, and rare peripheral/facial edema or asthma events; routine lab monitoring is generally not required [91, 92].

**We recommend the use of nemolizumab in patients with moderate or severe CPG.**

##### 3.3.3.9 Janus Kinase Inhibitors (JAKi)

JAKi, including abrocitinib, upadacitinib, and baricitinib, have demonstrated efficacy in CPG, likely by targeting its key inflammatory pathways, notably those involving type 2 cytokines such as IL‐4, IL‐13, and IL‐31. In an Italian multicenter real‐world study with 71 patients, the proportion of patients achieving a ≥ 4‐point reduction in peak pruritus at Week 16 was 100% for baricitinib, 94% for upadacitinib and 83% for abrocitinib, with sustained results at Week 24 [93]. This good response to JAKi in CPG is supported by several case series and case reports, some of which included patients who had previously not responded to dupilumab [8, 18, 94, 95, 96, 97, 98, 99, 100, 101, 102, 103, 104]. In a small multicentre retrospective cohort study with notable baseline differences between treatment groups, JAK1 inhibitors showed faster itch relief and greater lesion clearance signals than dupilumab [105]. However, in the absence of randomized controlled trials in CPG, [106] JAKi should be reserved for patients with high disease burden unresponsive to other systemic therapies. In cases associated with atopic dermatitis, in‐label use may be possible. JAKi carry regulatory safety warnings issued by the U.S. Food and Drug Administration (FDA), the European Medicines Agency (EMA), and Swissmedic, primarily based on the ORAL surveillance study of tofacitinib in patients with rheumatoid arthritis and predefined cardiovascular or malignancy risk factors [107]. To date, dermatology trials and real‐world data have not shown comparable safety signals in dermatologic indications. Specifically, a recent cohort study by Demirbas et al. involving 28 patients reported no serious adverse events or treatment discontinuations over 36 weeks, suggesting favorable short‐ to medium‐term safety [105]; however, as also emphasized by Vahabi et al., longer follow‐up is needed to better assess the potential long‐term risks of JAKi [106]. Thus, careful patient selection (e.g., avoidance in patients aged > 65 years), risk assessment and close monitoring remain mandatory [108, 109].

**We suggest the use of baricitinib, upadacitinib, or abrocitinib in CPG patients when other treatment options including approved biologics were unsatisfactory or contraindicated.**

##### 3.3.3.10 Retinoids, Neurokinin‐1 Receptor Antagonists, Thalidomide and Lenalidomide

Based on a review of the literature and our expert opinion, the available evidence is insufficient and/or the benefit‐tolerability profile is questionable to recommend the use of these drugs in CPG [69, 110, 111, 112, 113, 114], particularly in light of more effective and better‐tolerated alternative therapies.

**We cannot make a recommendation for the use of retinoids, neurokinin‐1 receptor antagonists, thalidomide, and lenalidomide.**

#### 3.3.4 Phototherapy

Narrow‐band UVB (NB‐UVB) phototherapy, and to a lesser extent ultraviolet A1 (UVA1) or psoralen plus ultraviolet A (PUVA), 3×/week for 4–12 weeks have proven effective treatment options for CPG. Evidence is derived from a limited number of prospective and retrospective studies as well as systematic reviews [74, 115, 116, 117, 118]. Relapse is common after discontinuation. NB‐UVB is both effective and very safe, making it particularly suitable for pregnant or breastfeeding patients. Limiting factors include the time commitment required and the lack of availability in some regions. Targeted 308‐nm excimer photo‐ or laser therapy has shown promise in small studies [119, 120, 121] and may be used for a few treatment‐resistant lesions. Combined with PUVA, excimer light can reduce cumulative UVA exposure [119].

**We recommend NB‐UVB (UVB311nm) as the preferred UV‐based modality in CPG. Alternatively, UVA1 or PUVA phototherapy may be used in selected cases; excimer lamp or laser treatment can be considered as an option for patients with a limited number of treatment‐resistant lesions.**

#### 3.3.5 Psychosomatic Treatment

Psychosomatic treatment plays an important role in CPG, as the condition is frequently associated with a high psychosocial burden and psychiatric comorbidities, including anxiety and depression, and less commonly obsessive‐compulsive disorder or attention‐deficit/hyperactivity disorder [9, 11, 122]. In addition, psychological factors such as stress, personality traits, and maladaptive behavioral patterns strongly influence itch perception and scratching behavior, thereby perpetuating the itch–scratch cycle [122, 123, 124, 125, 126].

Available evidence supports patient education regarding the close interaction between pruritus and psychological factors, as well as the use of cognitive‐behavioral therapy and relaxation techniques to improve coping strategies and modify scratching behavior [65, 122, 125]. Although psychosomatic interventions are recommended as part of a multidisciplinary treatment approach, robust evidence specifically addressing their efficacy in CPG remains limited. However, extrapolating from studies in pruritus populations other than CPG, as well as from our clinical experience, psychosomatic interventions may offer a modest additional benefit as adjunctive therapy [127, 128, 129].

**We suggest the use of adjunctive cognitive‐behavioral therapeutic techniques, particularly habit‐reversal training, in the management of CPG. This may include strategies aimed at relaxation and reduction of skin‐damaging scratching through conscious impulse control, distraction, or alternative behaviors (e.g., making fists or rubbing the skin with the back of the hand).**

## Conflicts of Interest

Antonio Cozzio has received honoraria as adviser or speaker for Abbvie, Almirall, BMS, Incyte, LEO Pharma, Eli Lilly, Janssen‐Cilag, Kiowa Kirin, Novartis, Pfizer, Sanofi, and UCB Pharma. Antonios G.A. Kolios has no COI related to this work. Ahmad Jalili has received honoraria as advisor or speaker, or received research grant or participated in clinical trials for AbbVie, Almirall, Amgen, Bayer, BioMed, Boehringer Ingelheim, Bristol‐Myers Squibb, Eli Lilly, Galderma, GlaxoSmithKline, Incyte Biosciences, Johnson & Johnson, La Roche‐Posay, LEO Pharma, Merz Pharma, MSD, Novartis, Pfizer, Sanofi Genzyme, Sandoz, Takeda, and UCB Pharma. Curdin Conrad has served as scientific adviser and/or clinical study investigator for AbbVie, Actelion, Almirall, Amgen, Boehringer Ingelheim, Bristol‐Myers Squibb, Celgene, Eli‐Lilly, Galderma, Incyte, Jiangsu Hengrui, Johnson & Johnson, LEO Pharma, MSD, Novartis, Pfizer, Samsung, Sanofi, Takeda, and UCB. Claudia Lang is a consultant and advisor and/or received speaking fees and/or grants and/or served as an investigator in clinical trials for AbbVie, Almirall, Amgen, ALK, Blueprint, Eli Lilly, Galderma, Incyte, Leo Pharma, Menarini AG, Novartis, Pfizer, Sanofi, and Thermo Fisher. Christoph Schlapbach has received honoraria as adviser or speaker for Abbvie, Almirall, BMS, GSK, Incyte, LEO Pharma, Lilly, Kiowa Kirin, Novartis, Pfizer, and Sanofi and has received research funding from PPM Services. Eva Loos received speaking fees for Sanofi‐Aventis AG and AbbVie AG. Helmut Beltraminelli was involved as advisor for Kyowa Kirin, Takeda, Amgen, Sanofi‐Aventis, Almirall. Maximilian Böhm has received honoraria as advisor and/or research or travel grants and/or served as an investigator in clinical trials for Sanofi‐Aventis AG, Boehringer Ingelheim, Boehringer Ingelheim Fonds, Incyte, Swiss Rockets AG, and Galderma SA. Peter Häusermann reports fees for Advisory Board and/or speaker honoraria and/or travel fees from AbbVie, Almirall, Eli Lilly, Galderma, Incyte, Janssen, Leo, Pfizer, Novartis, Sanofi, UCB. Simon Mueller was a consultant and advisor and/or received speaking fees and/or grants and/or served as an investigator in clinical trials for Sanofi‐Aventis AG, Galderma SA, Janssen‐Cilag AG, LEO Pharmaceutical Products Sarath, Amgen, Incyte, Novartis Pharma AG, and UCB Pharma. Wolf‐Henning Boehncke has no COI related to this work.

## Data Availability

Data sharing is not applicable to this article as no datasets were generated or analyzed during the current study.

## Appendix A

The literature search was conducted in PubMed to identify publications published after the release of the IFSI guideline (10/2020), allowing for a one‐year overlap. For each predefined therapeutic or thematic area, topic‐specific search terms (XY) were incorporated into the search strategy. The following search string was applied:

((“Prurigo Nodularis”[MeSH Terms] OR “prurigo nodularis”[Title/Abstract] OR “nodular prurigo”[Title/Abstract]).

OR (“Chronic Prurigo”[MeSH Terms] OR “chronic prurigo”[Title/Abstract])).

AND (“XY”[Title/Abstract]).

AND (“2019/01/01”[Date—Publication]: “2024/12/31”[Date—Publication]).
